# High thermal conductivity of high-quality monolayer boron nitride and its thermal expansion

**DOI:** 10.1126/sciadv.aav0129

**Published:** 2019-06-07

**Authors:** Qiran Cai, Declan Scullion, Wei Gan, Alexey Falin, Shunying Zhang, Kenji Watanabe, Takashi Taniguchi, Ying Chen, Elton J. G. Santos, Lu Hua Li

**Affiliations:** 1 Institute for Frontier Materials, Deakin University, Waurn Ponds, Victoria 3216, Australia.; 2 School of Mathematics and Physics, Queen’s University Belfast, Belfast BT7 1NN, UK.; 3 National Institute for Materials Science, Namiki 1-1, Tsukuba, Ibaraki 305-0044, Japan.

## Abstract

Heat management has become more and more critical, especially in miniaturized modern devices, so the exploration of highly thermally conductive materials with electrical insulation is of great importance. Here, we report that high-quality one-atom-thin hexagonal boron nitride (BN) has a thermal conductivity (κ) of 751 W/mK at room temperature, the second largest κ per unit weight among all semiconductors and insulators. The κ of atomically thin BN decreases with increased thickness. Our molecular dynamic simulations accurately reproduce this trend, and the density functional theory (DFT) calculations reveal the main scattering mechanism. The thermal expansion coefficients of monolayer to trilayer BN at 300 to 400 K are also experimentally measured for the first time. Owing to its wide bandgap, high thermal conductivity, outstanding strength, good flexibility, and excellent thermal and chemical stability, atomically thin BN is a strong candidate for heat dissipation applications, especially in the next generation of flexible electronic devices.

## Teaser

Atomically thin boron nitride is one of the best thermal conductors among semiconductors and insulators.

## INTRODUCTION

With increasing demand in miniaturization, thermal dissipation becomes critical for the performance, reliability, longevity, and safety of various products, such as electronic and optoelectronic devices, lithium ion batteries, and micromachines. Graphene has outstanding thermal transport: At near room temperature, the in-plane thermal conductivity (κ) of suspended graphene produced by mechanical exfoliation and chemical vapor deposition (CVD) was mostly in the range of 1800 to 5300 W/mK (*1*–*3*) and 1200 to 3100 W/mK (*4*–*10*), respectively. The electrical conductivity of graphene, however, prevents it from being directly used in many thermal dissipation applications, such as in electronics.

It is highly desirable to find electrical insulators with high thermal conductivities. It is well known that diamond and cubic boron nitride (cBN) are the best thermal conductors falling into this category. However, these two materials are expensive to produce because of the high temperature and pressure synthesis processes required. In addition, their brittleness makes them difficult to be incorporated into flexible devices. Very recently, high-quality cubic boron arsenide (BAs) with a bandgap of 1.5 eV was found to have a κ of ~1000 W/mK (*11*–*13*); however, it is unlikely to be flexible either. In comparison, the in-plane thermal conductivity of highly oriented pyrolytic hexagonal BN (HOPBN) was measured to be relatively small, i.e., ~400 W/mK at room temperature, although the HOPBN used in this early study consisted of small crystal domains (hence, many grain boundaries), defects, and dislocations (*14*).

Atomically thin BN is a relatively new form of hexagonal BN (hBN). It has a wide bandgap of ~6 eV that is not sensitive to thickness change (*15*) and is one of the strongest electrically insulating materials that is also highly flexible and stretchable (*16*). Atomically thin BN is an excellent dielectric substrate for graphene, molybdenum disulfide (MoS_2_), and many other two-dimensional (2D) material-based electronic and optical devices (*17*). In addition, the high thermal stability and impermeability of BN sheets are useful to passivate air-sensitive 2D materials and metal surfaces (*18*, *19*). The use of atomically thin BN in this aspect can be further extended to the coverage of plasmonic metal nanoparticles for surface-enhanced Raman spectroscopy, enabling much improved sensitivity, reproducibility, and reusability (*20*).

The thermal conductivity of monolayer (1L) BN has never been experimentally investigated, in spite of many theoretical studies (*21*–*24*). There have been experimental attempts on the κ of few-layer BN; however, most of the obtained values were less than that of bulk hBN. Jo *et al.* (*25*) reported the first experimentally derived κ of 5L and 11L BN that were ~250 and ~360 W/mK at room temperature, respectively. One year later, Zhou *et al.* (*26*) used Raman spectroscopy to find that the κ of CVD-grown 9L BN was in the range of 227 to 280 W/mK. Alam *et al.* and Lin *et al.* (*27*, *28*) also studied the κ of ~30-60L and few-layer CVD-grown BN. Wang *et al.* (*29*) measured a 2L BN using prepatterned thermometers and deduced a κ of 484 + 141/−24 W/mK at room temperature. The thickness effect has only been reported by Jo *et al.* (*25*) who found that 5L BN had a worse heat-spreading property than 11L BN. This was opposite to the trend observed in graphene where κ dropped from ~4000 to ~2800 and ~1300 W/mK from one to two and four layers, respectively (*30*).

Therefore, it is still an open question whether atomically thin BN has higher κ values than bulk hBN and how the thickness affects its κ. Single-crystalline and surface-clean mono- and few-layer BN samples are needed to reveal their intrinsic κ and the thickness effect. In the case of graphene, the crystal quality and surface cleanness could markedly affect its κ (*6*, *9*). In the aforementioned studies of few-layer BN, the samples were either mechanically exfoliated from imperfect (commercial) hBN powder or grown by CVD. Furthermore, polymer transfer processes involving either poly(methyl methacrylate) (PMMA) or polydimethylsiloxane were used in all these studies to prepare suspended BN, which inevitably left polymer residues. These polymer residues caused strong phonon scattering in graphene as well as in atomically thin BN due to their atomic thickness (*25*). On the other hand, thermal expansion is a fundamental property of any material, which is important to material processing and application. There still lacks the experimental examination of the thermal expansion coefficients (TECs) of atomically thin BN.

Here, we report the thermal conductivity coefficients, TECs of high-quality single-crystalline atomically thin BN without polymer contamination. According to optothermal Raman measurements, the suspended 1L BN had a high average κ of 751 W/mK at close to room temperature, and therefore, it was one of the best thermal conductors among semiconductors and electrical insulators. The κ of 2-3L BN dropped to 646 and 602 W/mK, respectively. Molecular dynamic (MD) and density functional theory (DFT) simulations were used to gain insights into the thickness effect on the κ of atomically thin BN. In addition, we experimentally revealed that 1-3L BN had negative TECs in the range of −3.58 × 10^−6^/K and − 0.85 × 10^−6^ /K at 300 to 400 K.

## RESULTS

**Fig. 1:**
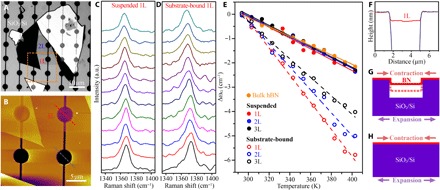
The first-order temperature coefficients. (**A**) Optical image of a 1-2L BN on SiO_2_/Si substrate with prefabricated microwells. (**B**) AFM image of the squared area in (A). (**C** and **D**) Raman *G* bands of the 1L BN suspended over and bound to SiO_2_/Si at different heating stage temperatures from 293 to 403 K with an interval of 10 K. a.u., arbitrary units. (**E**) Summarized *G* band frequency changes of the suspended and substrate-bound 1-3L BN as a function of temperature and the corresponding linear fittings. (**F**) AFM height traces of the dash lines in (B). (**G** and **H**) Schematic diagrams of the thermal expansion of suspended and substrate-bound BN nanosheets.

We used the Raman technique to measure the κ of high-quality and clean atomically thin BN, as previously used for graphene (*1*, *3*–*5*, *26*). Atomically thin BN flakes were mechanically exfoliated from hBN single crystals (*31*) using Scotch tape onto two different substrates: silicon covered by 90-nm oxide layer (SiO_2_/Si) and 80-nm gold-coated silicon (Au/Si), both with prefabricated microwells (diameter, 3.8 μm) and connecting trenches (width, 0.2 μm). The trenches acted as vents to avoid trapped air in BN-covered microwells from expansion during heating. According to our previous studies, the BN sheets prepared by this method were almost free of defects and grain boundaries (*16*, *18*). The absence of a polymer-based transfer process prevented surface contamination that could deteriorate thermal conductivity. An optical microscope was used to locate atomically thin sheets followed by atomic force microscopy (AFM) to determine their thickness. Figure 1 (A and B) shows the optical and AFM images of a 1L BN with a thickness of 0.48 nm on SiO_2_/Si. The Raman spectra of the suspended 1-3L and bulk BN are compared in fig. S2.

For temperature coefficients, we studied the temperature-dependent Raman spectra of three different 1-3L BN samples: suspended over SiO_2_/Si, bound to SiO_2_/Si, and suspended over Au/Si using a relatively small laser power of 0.84 to 1.63 mW to minimize the heating effect. Atomically thin BN bound to Au/Si showed Raman signals that are too weak to be useful and hence was excluded from the study. Figure 1 (C and D) displays the typical Raman spectra of 1L BN suspended over and bound to SiO_2_/Si at 293 to 403 K, respectively, and the Raman shifts of 1-3L and bulk hBN are summarized in Fig. 1E. Linear fittings, i.e., ω − ω_0_ = χ*T*, were applied to estimate the first-order temperature coefficients (χ), where ω − ω_0_ is the change of the *G* band frequency due to temperature variation and *T* is temperature. The suspended 1-3L BN showed quite similar χ: −0.0223 ± 0.0012, −0.0214 ± 0.0010, and −0.0215 ± 0.0007 cm^−1^/K, respectively, quite close to that of the suspended bulk hBN single crystals, i.e., −0.0191 ± 0.0005 cm^−1^/K. In contrast, those of the substrate-bound 1-3L flakes were very different: −0.0558 ± 0.0011, −0.0480 ± 0.0022, and − 0.0380 ± 0.0011 cm^−1^/K, respectively.

The observed frequency downshifts with increased temperature could be caused by three factors: (i) the thermal expansion of BN lattice ($\Delta \omega_{G}^{E}$); (ii) anharmonic phonon-phonon effects ($\Delta \omega_{G}^{A}$); and (iii) the TEC mismatch between BN sheets and the SiO_2_/Si substrate. As atomically thin BN sheets are insulators, substrate doping was negligible (*32*). In principle, these three effects should be exactly the same for the 1-3L BN no matter suspended over, or bound to SiO_2_/Si. That is, the SiO_2_/Si substrate with or without microwells should expand the same amount with the same temperature increase. However, our results in Fig. 1E told a different story. It has been reported that mechanically exfoliated atomically thin materials, e.g., graphene, tend to partially adhere to the side wall of microwells via van der Waals attraction (*33*). Our AFM results verified the existence of this phenomenon in our suspended atomically thin BN. For example, the AFM height trace of the 1L BN in Fig. 1 (A and B) showed that it was 29.2 nm below the surface of the substrate (Fig. 1F). We believe that the hanging-down gave the suspended atomically thin BN the capability to eliminate the third effect during heating, i.e., the TEC mismatch between BN sheets and the substrate. That is, the suspended atomically thin BN could peel off or adhere more to the side walls with minimum energy dissipation to fully relax and accommodate the strain owing to the TEC mismatch (Fig. 1G). This proposition was strongly backed up by our measured Raman shifts of 1-3L BN suspended over Au/Si. The TEC of Au is >30 times that of SiO_2_ at close to room temperature, which should give rise to markedly more influence from the TEC mismatch effect. However, the 1-3L BN suspended over Au/Si showed very similar fitting slopes to those of the samples suspended over SiO_2_/Si (fig. S3). Therefore, the intrinsic χ of 1-3L BN could be obtained from the linear fittings of the temperature-dependent Raman shifts of the BN sheets suspended over SiO_2_/Si. The values from samples suspended over Au/Si, though close, were not used further because of the much larger TEC of Au and its potentially detrimental effect on the accuracy of χ. On the other hand, the different temperature-dependent Raman shifts of 1-3L BN bound to SiO_2_/Si suggested different TECs of atomically thin BN, which will be discussed in detail later.

**Fig. 2:**
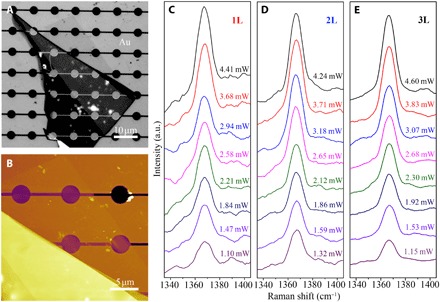
Laser power effect. Optical (**A**) and AFM (**B**) images of a 1L BN on Au/Si. The Raman *G* bands of the suspended (**C**) 1L BN, (**D**) 2L BN, and (**E**) 3L BN under different laser power. a.u., arbitrary units.

Next, the effect of laser heating on the Raman frequency of 1-3L BN suspended over Au/Si was investigated. The Au film with a much higher κ than SiO_2_ performed as a heat sink kept at room temperature during the measurements. Figure 2 (A and B) shows the optical and AFM images of a 1L BN with a thickness of 0.52 nm covering four microwells in Au/Si. Figure 2 (C to E) exemplifies the Raman spectra of the *G* bands of suspended 1-3L BN under different laser power. Raman downshifts were observed in all samples, suggesting increased local temperature with incremental laser power. However, such temperature increase was far from dramatic (i.e., ~25 K). The laser-induced Raman frequency change of the suspended atomically thin BN sheets correlated to their capabilities of thermal conduction to the edge of the microwells, i.e., heat sink. With the heat loss into the ambient (via air and radiation) taken into account, the temperature distribution *T*(*r*) in the suspended BN can be written as (*4*)

$$T(r)=T_{1}+\frac{Q-Q_{\text{air}}}{2\pi d\kappa }\text{ln}(\frac{R}{r})\beta (r),r\leq R$$ (1)

where *T*_1_ is the temperature at the edge of the suspended BN, i.e., the boundary condition, *T*(*R*) = *T*_1_; *Q* is the absorbed laser power; *Q*_air_ is the heat loss into the air; *d* is BN thickness; κ is thermal conductivity; and *R* is the radius of the microwell (1.9 μm).

**Fig. 3:**
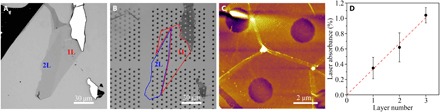
Light absorbance of atomically thin BN. Optical images of a 1-2L BN as exfoliated on SiO_2_/Si (**A**) and transferred onto a Si_3_N_4_ TEM grid (**B**). (**C**) AFM image of the BN suspended over the TEM grid. (**D**) Laser absorbance of 1-3L BN and the corresponding linear fitting.

It was reported that light absorption could greatly affect the accuracy of Raman-deduced thermal conductivity (*3*, *4*). The total laser power absorbed by the BN (*Q*) equals to the multiplication of the light absorbance (*I*_ab_) with the laser power (*P*). We tried three methods to accurately determine the light absorbance of 1-3L BN at 514.5 nm. (i) We used PMMA to transfer atomically thin BN from SiO_2_/Si onto silicon nitride (Si_3_N_4_) transmission electron microscopy (TEM) grids with patterned 2-μm holes. The polymer was removed by annealing at 550°C in air. Figure 3 (A to C) shows the optical and AFM images of a 1-2L BN before and after the transfer. The absorbance values of 1-3L BN were 0.35 ± 0.14%, 0.62 ± 0.19%, and 1.04 ± 0.10%, respectively, measured by an optical power meter (Fig. 3D). These values closely followed the linear dashed line across the (0, 0) origin. (ii) We also transferred atomically thin BN onto a transparent quartz plate by PMMA. The absorbance of BN sheets could be estimated by deduction of the light absorption of the quartz without consideration of the weak light reflection of 2D sheets (*34*). The absorbance values of 1-3L BN deduced from linear fitting were 0.34 ± 0.02%, 0.67 ± 0.03%, and 1.01 ± 0.04%, respectively (fig. S4). (iii) We used transmitted optical microscopy under visible light, and the absorbance of 1L BN was ~0.4 to 0.5% (fig. S5) in reasonable agreement with the value measured by the optical power meter in the other two methods. For the calculation of κ, we used the absorbance values from the first method. These values were only ~15% of those measured from graphene and much smaller than those used in previous calculations of the thermal conductivity of few-layer BN. Zhou *et al.* (*26*) measured the absorbance of 1-2L and 9L CVD-grown BN transferred onto glass slides, and the values were 1.5 and 5.1% at 514.5-nm wavelength, respectively. Lin *et al.* (*28*) obtained an absorbance of ~3% for a 2.1-nm-thick (6L) CVD-grown BN transferred to a quartz substrate. The small absorbance that we obtained is reasonable if one considers the wide bandgap (i.e., ~6 eV) of high-quality BN, which should have minimal light absorption at the wavelength of ~500 nm; however, defect states can markedly increase its light absorption in the visible range. So, the small temperature increases of the atomically thin BN with the increase of laser power (Fig. 2) were mainly due to its weak absorption of the 514.5-nm light

$$Q_{\text{air}}=\int_{r_{0}}^{R}2\pi h(T-T_{a})\mathrm{rdr}+\pi r_{0}^{2}h(T_{m}-T_{a})$$ (2)

where *r*_0_ is the radius of the laser beam, which was estimated to be 0.31 ± 0.01 μm by performing a Raman line scan of the edge of the Au-covered Si wafer (fig. S6) (*4*); *h* is the heat transfer coefficient of hBN. In the case of a small temperature difference between an object and the ambient, the quadratic expression for radiation can be linearized to reach the total heat transfer coefficient as the sum of convective(*h*_c_) and radiative components. Therefore, *h* = *h*_c_ + εσ4*T*^3^, where *h*_c_ = 3475 W/m^2^K for BN sheets and ε is the emissivity (0.8 for hBN), and σ = 5.670373 × 10^−8^ W/m^2^K^4^ is the Stefan-Boltzmann constant (*27*)

$$\beta (r)=1+\frac{\mathrm{Ei}(-\frac{r^{2}}{r_{0}^{2}})-\mathrm{Ei}(-\frac{R^{2}}{r_{0}^{2}})}{2\text{ln}(\frac{R}{r})}$$ (3)

Note that *r*_0_ was much smaller than the radius of the microwells. The Raman-measured temperature (*T*_m_) of the suspended BN can be estimated by

$$T_{m}\approx \frac{\int_{0}^{R}T(r)\text{exp}(-\frac{r^{2}}{r_{0}^{2}})r\;\mathrm{dr}}{\int_{0}^{R}\text{exp}(-\frac{r^{2}}{r_{0}^{2}})r\;\mathrm{dr}}$$ (4)

Given that the thermal resistance between the atomically thin BN and Au heat sink was negligible because of the relatively large contact area and high κ of Au, the thermal conductivity of the suspended BN can be approximated as

$$\kappa =\frac{\text{ln}(\frac{R}{r_{0}})}{2\pi d\frac{T_{m}-T_{a}}{Q-Q_{\text{air}}}}\alpha$$ (5)

where α is the Gaussian profile factor of the laser beam

$$\alpha =\frac{T_{m}-T_{1}}{T_{0}-T_{1}}\beta (r_{0})$$ (6)

where *T*_0_ is the temperature of the suspended BN at a radial distance of *r*_0_. In our experimental setup, $\frac{T_{m}-T_{1}}{T_{0}-T_{1}}$ is ~1.03 and β(*r*_0_) is ~0.94, so α is 0.97. *T*_a_ is the ambient temperature (298 K); $\frac{T_{m}-T_{a}}{Q-Q_{\text{air}}}$ denotes the increased temperature at the center of the suspended BN due to the absorbed laser power and can be deduced from Fig. 2 (C to E).

**Fig. 4:**
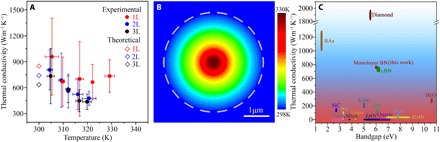
Thermal conductivity of 1-3L BN. (**A**) Experimental κ of the suspended 1-3L BN as a function of temperature (filled circles) and the corresponding theoretical values at 300 K (open rhombus). (**B**) Temperature distribution of a suspended 1L BN over 3.8-μm microwells under laser heating up to 330 K with the heat sink kept at 298 K, and the dashed circle represents the edge of the suspended BN. (**C**) Comparison of the thermal conductivity of some common semiconductors and insulators.

The thermal conductivities of the suspended 1-3L BN as a function of the measured temperature *T*_m_ were plotted in Fig. 4A (circles). The unusual temperature-dependent κ of 1L BN should be due to the uncertainty in the optothermal measurements, especially its low optical absorption and hence small temperature change. Therefore, we averaged the κ of 1-3L BN at close to room temperature (based on a total of 12 samples), and their values were 751 ± 340, 646 ± 242, and 602 ± 247 W/mK, respectively. It should also be noted that the optothermal method ignores nonequilibrium in different phonon polarizations, which leads to underestimated κ. The error was calculated through the root sum square error propagation approach, where the temperature calibration by Raman, the temperature resolution of the Raman measurement, and the uncertainty of the measured laser absorbance were considered (Supplementary Materials). For 1L BN, the heat loss to air (*Q*_air_) only accounted for ~2.6% of the total heat dissipation during laser heating, and the values were even smaller for 2L and 3L BN (1.4 and 1.0%, respectively). We also used the same procedure to measure the κ of a 1L graphene exfoliated from highly oriented pyrolytic graphite (HOPG), which gave a value of 2102 ± 221 W/mK (fig. S7). The thermal conductivities of some common semiconductors and insulators as a function of their bandgaps are compared in Fig. 4C. One-atom-thin BN is the third most thermally conductive among semiconductors and insulators, just behind diamond and cBAs. It should also be mentioned that the thermal conductivity per unit weight of a material is important for its application, e.g., in portable devices. hBN has a density of 2.2 g/cm^3^, smaller than that of cBAs (5.2 g/cm^3^) and cBN (3.4 g/cm^3^). That is, 1L BN has the second largest thermal conductivity per unit weight, just behind diamond.

**Fig. 5:**
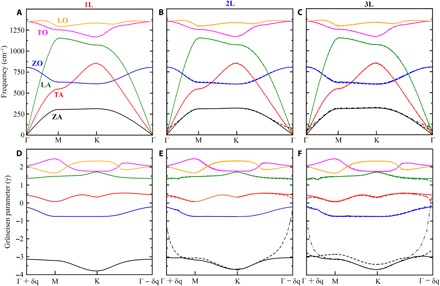
Phonon dispersion and Grüneisen parameters. (**A** to **C**) Phonon dispersion and (**D** to **F**) Grüneisen parameters of 1-3L BN calculated by DFT. The phonon branches are labeled for 1L BN. Dashed curves represent additional phonon branches and corresponding Grüneisen parameters due to additional BN layers.

MD calculations (see Materials and Methods for details) were conducted on the thermal conductivity of atomically thin BN. The obtained theoretical κ of 849, 740, and 634 W/mK for 1-3L BN at ~300 K, respectively (Fig. 4A, open rhombus) were in line with the experimental values. Our calculated value of 1L BN was close to that reported by Lindsay *et al.* (*35*) by considering a 10-μm grain size. The experimental trend that the κ of BN decreased with increased thickness was also observed in the simulations. To explain this, the phonon dispersion and Grüneisen parameters of 1-3L BN were calculated by DFT (Fig. 5). There were three optical branches, namely, longitudinal optical (LO), transverse optical (TO), and out-of-plane optical (ZO) modes, and three acoustic branches, namely, longitudinal acoustic (LA), transverse acoustic (TA), and out-of-plane acoustic (ZA) branches. Similar to graphene, the LO, TO, and ZO branches hardly contributed to the thermal conductivity of 1-3L BN, and the ZA contribution was far larger than those from TA and LA (*30*, *36*). Any additional layers added to 1L BN created more ZA phonon states (Fig. 5, A to C) available for Umklapp scattering, which was the dominating limitation in the thermal conductivity of defect- and grain boundary–free and surface-clean few-layer BN. Furthermore, the Grüneisen parameter and phonon frequency of the ZA mode increased with additional BN layers (Fig. 5, D to F), which was further evidence that stronger Umklapp scattering occurred in few-layer BN as

$$\frac{1}{\tau }=2\gamma^{2}\frac{k_{B}T\omega }{{\mathrm{Mv}}^{2}\omega_{m}}$$ (7)

where τ is the intrinsic phonon relaxation time associated with phonon-phonon Umklapp scattering; γ is Grüneisen parameter; *M* is the atomic mass; ω*_m_* is the Debye frequency; *T* is the temperature; *k*_B_ is the Boltzman constant; and *v* is the averaged sound velocity. Larger Grüneisen parameters and phonon frequency of the ZA mode led to shorter relaxation time and more phonon scattering. The trend we observed from atomically thin BN that its κ increased with decreasing layer thickness was also found on graphene and MoS_2_, whose thermal conductivities increased from 1300 to 2800 W/mK and from 98 to 138 W/mK, respectively, when their thickness decreased from 4 to 2L and 3 to 1L, respectively (*37*, *38*). It is reasonable to believe that this unusual thickness effect on thermal conductivity is intrinsic for 2D materials.

**Fig. 6:**
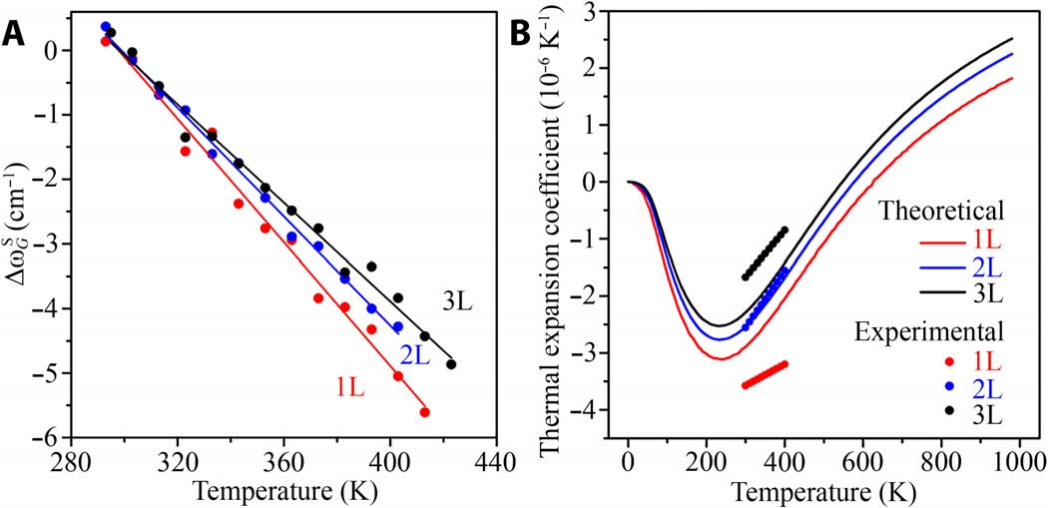
TECs of 1-3L BN. (**A**) The *G* band frequency shifts as a function of temperature and the corresponding fittings of 1-3L BN bound to SiO_2_/Si using TECs as fitting parameters. (**B**) Experimental (dots) and theoretical (lines) curves of the TECs of the 1-3L BN.

As aforementioned, we could also use temperature-dependent Raman spectroscopy to estimate the TECs of atomically thin BN when it is bound to the SiO_2_/Si substrate, as the effect of TEC mismatch between the BN and the substrate was included. The temperature-dependent *G* band shift (Δω*_G_*) of substrate-bound BN nanosheets could be written as

$$\Delta \omega_{G}=\Delta \omega_{G}^{E}(T_{m})+\Delta \omega_{G}^{A}(T_{m})+\Delta \omega_{G}^{S}(T_{m})$$ (8)

where *T*_m_ is the measured temperature of the sample; $\Delta \omega_{G}^{E}(T_{m})$ is the thermal expansion of atomically thin BN; $\Delta \omega_{G}^{A}(T_{m})$ is anharmonic effect; and $\Delta \omega_{G}^{S}(T_{m})$ is the effect of the strain ε(*T*_m_) due to the TEC mismatch between atomically thin BN and the SiO_2_/Si, which can be expressed as

$$\Delta \omega_{G}^{S}(T_{m})=\beta \varepsilon (T_{m})=\beta \int_{297}^{T_{m}}(\alpha_{{\text{SiO}}_{2}/\text{Si}}(T)-\alpha_{\text{BN}}(T))\mathrm{dT}$$ (9)

where β is the biaxial strain coefficient of the *G* band of atomically thin BN. β = 2γω_o_ and γ is the Grüneisen parameters of 1-3L BN (Fig. 5, D to F), and ω_o_ is the strain-free *G* band frequency (*39*). Therefore, β values for 1-3L BN are −56.07, −56.03, and − 55.99 cm^−1^/% for 1-3L BN, respectively. α_SiO_2__/Si__ and α_BN_ are the temperature-dependent TECs of 90-nm SiO_2_/Si and BN sheets, respectively. We used finite element method (FEM) to accurately calculate α_SiO_2__/Si__ in the temperature range of 300 to 400 K (fig. S9). Because the temperature-dependent *G* band shifts of the suspended BN nanosheets were contributed only by the thermal expansion of atomically thin BN lattice ($\Delta \omega_{G}^{E}$) and anharmonic effects ($\Delta \omega_{G}^{A}$), we can use Eq. 9, i.e., the TECs of BN nanosheets as variants, to fit the experimental data of the *G* band shifts of the substrate-bound BN nanosheets (Fig. 6A). The TECs of 1-3L BN were estimated to be (−3.58 ± 0.18) × 10^−6^, (−2.55 ± 0.28) × 10^−6^, and (−1.67 ± 0.20) × 10^−6^/K at room temperature, close to those of bulk hBN and graphene (*39*, *40*). The TECs of atomically thin BN were also calculated by DFT, and the theoretical and experimental curves are compared in Fig. 6B. The difference between the two could be due to the limitation of the exchange-correlation functional in representing the fundamental vibrational modes, as we have pointed out recently (*41*). Atomically thin BN has the smallest TECs among the commonly studied 2D materials (Supplementary Materials).

## DISCUSSION

In summary, suspended high-quality and surface-clean one-atom-thin and few-layer BN sheets were prepared to reveal their intrinsic κ. The Raman-deduced average κ for 1-3L BN were 751 ± 340, 646 ± 242, and 602 ± 247 W/mK at room temperature, respectively. The trend that the κ decreased with increased thickness was caused by the interlayer interaction, resulting in more phonon branches and states available for Umklapp scattering in few-layer BN. We also experimentally investigated the TECs of atomically thin BN: (−3.58 ± 0.18) × 10^−6^, (−2.55 ± 0.28) × 10^−6^, and (−1.67 ± 0.20) × 10^−6^/K for 1-3L BN at close to room temperature, respectively. This study contributes to current knowledge of the thermal conductivity of 2D materials and shows that atomically thin BN sheets have better thermal conductivity than bulk hBN as well as most of semiconductors and insulators, except diamond and cBAs. Along with its low density, outstanding strength, high flexibility and stretchability, good stability, and excellent impermeability, atomically thin BN is a promising material for heat dissipation in different applications.

## MATERIALS AND METHODS

### Sample preparation and Raman measurement

The trench-connected microwells in a Si wafer were fabricated by the combination of photolithography and electron beam lithography. The depth for both the microwells and trenches was ~2 μm. A metal sputter (EM ACE600, Leica) was used to coat the Au film that served as a heat sink. The suspended atomically thin BN sheets were mechanically exfoliated on the Au/Si and SiO_2_/Si from hBN single crystals. The optical microscope and AFM were Olympus BX51 and Asylum Research Cypher. A Renishaw inVia micro-Raman system equipped with a 514.5-nm laser was used. In all experiments, a 100× objective lens with a numerical aperture of 0.90 was used. All Raman spectra were calibrated with the Raman band of Si at 520.5 cm^−1^. The laser power passing the objective lens was measured by an optical power meter (1916-C, Newport). A heating stage (LTS350, Linkam) was used for temperature control.

### MD using classical potentials

Thermal conductivity coefficients κ were calculated using the Green-Kubo approach (*42*), which was simulated by the integration of the time-dependent heat-flux autocorrelation functions via

$$\kappa_{\alpha \beta }=\frac{1}{k_{B}T^{2}V}\int_{0}^{\infty }\langle J_{\alpha }(t)J_{\beta }(0)\rangle \mathrm{dt}$$ (10)

where *t* is the time; *T* and *V* are the system temperature and volume, respectively; and *J*_α, β_ are the components of the lattice heat current vector $\vec{J}$ along the α and β components. 〈*J*_α_(*t*)*J*_β_(0)〉 is the ensemble averaged heat current autocorrelation function. In this work, α = β because of the symmetry of the hBN lattice along the in-plane. The heat current vector is defined as

$$\vec{J}(t)=\frac{d}{\mathrm{dt}}\sum_{i}{\vec{R}}_{i}E_{i}=\sum_{i}E_{i}{\vec{v}}_{i}+\sum_{i}\frac{{\mathrm{dE}}_{i}}{\mathrm{dt}}{\vec{R}}_{i}$$ (11)

where $\vec{R_{i}}$, *v_i_*, and *E_i_* are the position, velocity, and the energy of atom *i*, respectively. Calculations were carried out within MD simulations using Large-scale Atomic/Molecular Massively Parallel Simulator (LAMMPS). The three-body Tersoff potential (*43*) was used to treat the in-plane interactions, and a Lennard-Jones potential was used to treat the out-of-plane interactions. The parameters of these potentials have been described elsewhere (*35*). The DFT-relaxed structures were used as an initial guess and then further minimized within LAMMPS. The system was then equilibrated under an NVT ensemble for 2.5 ns at 300 K. Following this, the Green-Kubo method was used to calculate the κ. Calculations were run under an NVE ensemble for 10 ns with a time step of 0.5 fs (fig. S8). The simulated κ values converged within ~5.5 ns, after which the κ magnitudes were averaged and used to determine the final κ reported in this work. Different correlation lengths *p* of 400, 500, and 600 ps with a sampling interval *s* of 10 ps were used along with an effective volume of N_x_*2.50*N_y_*4.33*N_L_*3.33 and 36,000 atoms in the calculation of the thermal conductivity. *N_x_* and *N_y_* are the numbers of unit cells in the *x* and *y* directions respectively, and *N*_L_ is the number of layers.

### Ab initio DFT

Theoretical calculations were carried out within DFT using the Vienna Ab initio Simulation Package (VASP) (*44*). The generalized gradient approximation (*45*) along with many-body dispersion force corrections (*46*, *47*) was used along with a well-converged 800-eV plane wave cutoff. The projector augmented-wave (*48*) pseudopotentials were used to model the bonding environments of B and N. The atomic positions and lattice vectors were allowed to relax until the forces on the atoms and pressure on the cell were less than 0.000005 eV/Å and 0.01 GPa, respectively. A 24 × 24 × 1 Γ-centered k-grid was used to sample the Brillouin zone. TECs were calculated using the Phonopy code (*49*) and the quasi-harmonic approximation. A 2 × 2 × 1 supercell was used in all phonon calculations.

## Supplementary Material

http://advances.sciencemag.org/cgi/content/full/5/6/eaav0129/DC1

Download PDF

## SUPPLEMENTARY MATERIALS

Supplementary material for this article is available at http://advances.sciencemag.org/cgi/content/full/5/6/eaav0129/DC1 Section S1. Optical and AFM images of atomically thin BN samples Section S2. Raman spectra of the suspended 1-3L and bulk BN Section S3. Temperature coefficients of the 1-3L BN suspended over Au/Si substrate Section S4. Absorbance of 1-3L BN measured on quartz Section S5. Laser beam radius Section S6. Error calculation Section S7. Thermal conductivity of graphene as a control Section S8. Thermal equilibration on MD simulations using LAMMPS Section S9. TEC of SiO_2_/Si substrate simulated by FEM Section S10. Comparison of the TEC of common 2D materials Fig. S1. Characterizations of additional 1-3L BN. Fig. S2. Raman *G* bands of 1-3L and bulk BN. Fig. S3. Raman *G* band shifts of 1-3L BN suspended over Au/Si and SiO_2_/Si as a function of temperature and the corresponding linear fittings. Fig. S4. Laser absorbance of atomically thin BN on quartz. Fig. S5. Transmitted optical intensity of 1L BN. Fig. S6. Raman mapping of Si and corresponding fitting. Fig. S7. The first-order temperature coefficient and thermal conductivity of graphene. Fig. S8. Temperature versus time step for 1L BN. Fig. S9. Strain distribution of SiO_2_/Si substrate. Table S1. TEC of 2D materials (10^−6^ K^−1^).
