# Supplementary material for: High thermal conductivity of high-quality monolayer boron nitride and its thermal expansion
Source: Sci Adv. 2019 Jun 7;5(6):eaav0129. doi: 10.1126/sciadv.aav0129 (PMC6555632; doi:10.1126/sciadv.aav0129)
Supplement: http://advances.sciencemag.org/cgi/content/full/5/6/eaav0129/DC1 [file supp_5_6_eaav0129__index.html]

Science Advances | Science Advances

## Supplementary Materials

**This PDF file includes:**

- Section S1. Optical and AFM images of atomically thin BN samples
- Section S2. Raman spectra of the suspended 1-3L and bulk BN
- Section S3. Temperature coefficients of the 1-3L BN suspended over Au/Si substrate
- Section S4. Absorbance of 1-3L BN measured on quartz
- Section S5. Laser beam radius
- Section S6. Error calculation
- Section S7. Thermal conductivity of graphene as a control
- Section S8. Thermal equilibration on MD simulations using LAMMPS
- Section S9. TEC of SiO2/Si substrate simulated by FEM
- Section S10. Comparison of the TEC of common 2D materials
- Fig. S1. Characterizations of additional 1-3L BN.
- Fig. S2. Raman *G* bands of 1-3L and bulk BN.
- Fig. S3. Raman *G* band shifts of 1-3L BN suspended over Au/Si and SiO2/Si as a function of temperature and the corresponding linear fittings.
- Fig. S4. Laser absorbance of atomically thin BN on quartz.
- Fig. S5. Transmitted optical intensity of 1L BN.
- Fig. S6. Raman mapping of Si and corresponding fitting.
- Fig. S7. The first-order temperature coefficient and thermal conductivity of graphene.
- Fig. S8. Temperature versus time step for 1L BN.
- Fig. S9. Strain distribution of SiO2/Si substrate.
- Table S1. TEC of 2D materials (10−6 K−1).

Download PDF

**Files in this Data Supplement:**

- Adobe PDF - aav0129\_SM.pdf
