# Supplementary material for: High thermal conductivity of high-quality monolayer boron nitride and its thermal expansion
Source: Sci Adv. 2019 Jun 7;5(6):eaav0129. doi: 10.1126/sciadv.aav0129 (PMC6555632; doi:10.1126/sciadv.aav0129)
Supplement: Download PDF [file aav0129_SM.pdf]

Science Advances
AAAS

advances.sciencemag.org/cgi/content/full/5/6/eaav0129/DC1

Supplementary Materials for

High thermal conductivity of high-quality monolayer boron nitride and its thermal expansion

Qiran Cai, Declan Scullion, Wei Gan, Alexey Falin, Shunying Zhang, Kenji Watanabe, Takashi Taniguchi, Ying Chen, Elton J. G. Santos*, Lu Hua Li*

*Corresponding author. Email: luhua.li@deakin.edu.au (L.H.L.); e.santos@qub.ac.uk (E.J.G.S.)

Published 7 June 2019, *Sci. Adv.* **5**, eaav0129 (2019)
DOI: 10.1126/sciadv.aav0129

This PDF file includes:

Section S1. Optical and AFM images of atomically thin BN samples
Section S2. Raman spectra of the suspended 1-3L and bulk BN
Section S3. Temperature coefficients of the 1-3L BN suspended over Au/Si substrate
Section S4. Absorbance of 1-3L BN measured on quartz
Section S5. Laser beam radius
Section S6. Error calculation
Section S7. Thermal conductivity of graphene as a control
Section S8. Thermal equilibration on MD simulations using LAMMPS
Section S9. TEC of SiO_2/Si substrate simulated by FEM
Section S10. Comparison of the TEC of common 2D materials
Fig. S1. Characterizations of additional 1-3L BN.
Fig. S2. Raman *G* bands of 1-3L and bulk BN.
Fig. S3. Raman *G* band shifts of 1-3L BN suspended over Au/Si and SiO_2/Si as a function of temperature and the corresponding linear fittings.
Fig. S4. Laser absorbance of atomically thin BN on quartz.
Fig. S5. Transmitted optical intensity of 1L BN.
Fig. S6. Raman mapping of Si and corresponding fitting.
Fig. S7. The first-order temperature coefficient and thermal conductivity of graphene.
Fig. S8. Temperature versus time step for 1L BN.
Fig. S9. Strain distribution of SiO_2/Si substrate.
Table S1. TEC of 2D materials (10^{-6} K^{-1}).

Section S1. Optical and AFM images of atomically thin BN samples

Figure S1 shows additional optical and atomic force microscopy (AFM) images of the mechanically exfoliated 1-3L BN suspended over pre-fabricated microwells.

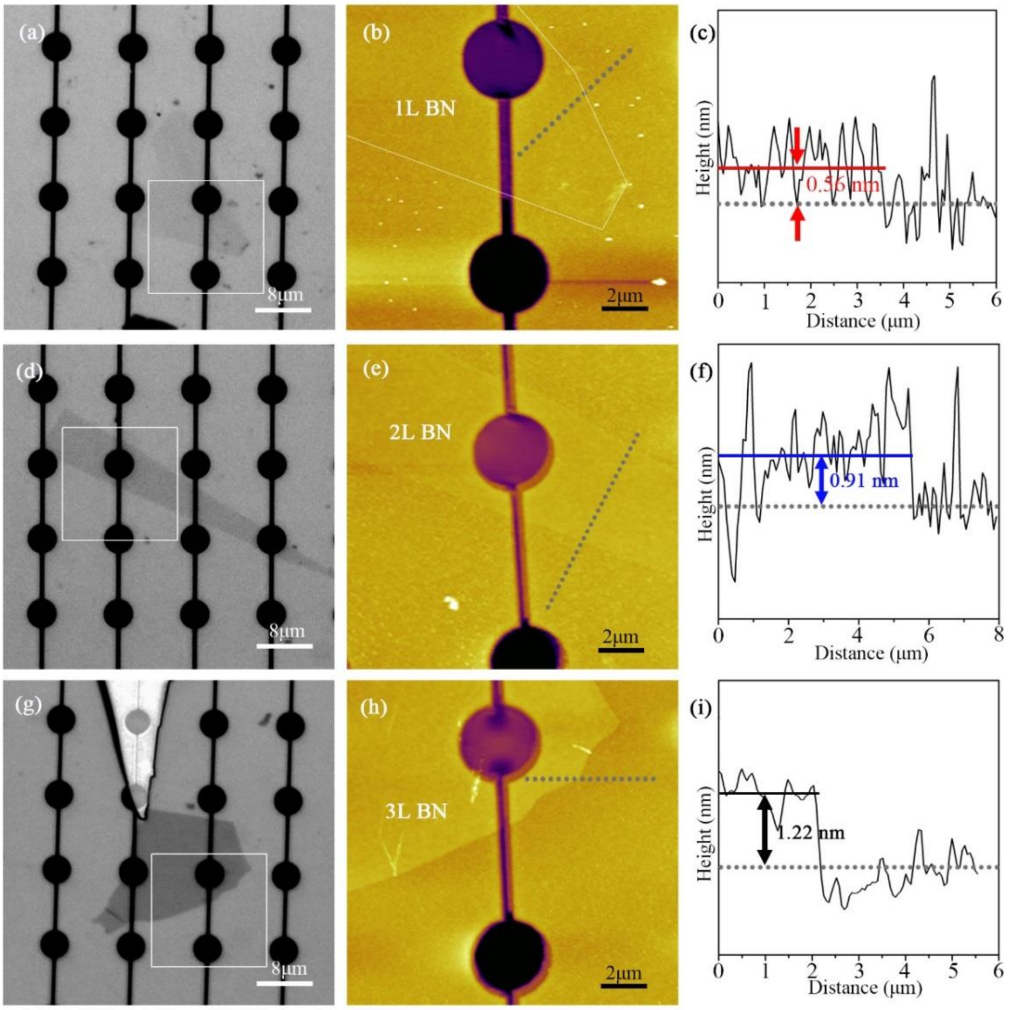

Fig. S1. Characterizations of additional 1-3L BN. (a-i) Optical microscopy photos, AFM images, and the corresponding height traces of additional 1-3L BN.

Section S2. Raman spectra of the suspended 1-3L and bulk BN

The Raman G bands of the suspended 1-3L and bulk BN are compared in fig. S2.

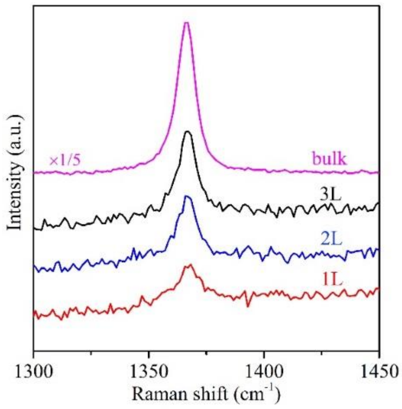

Fig. S2. Raman *G* bands of 1-3L and bulk BN.

Section S3. Temperature coefficients of the 1-3L BN suspended over Au/Si substrate

The temperature-dependent Raman G band shifts and corresponding fittings of 1-3L BN suspended over 90 nm oxide layer (SiO_2/Si) and 80 nm gold-coated silicon (Au/Si) are summarized and compared in fig. S3. For Au/Si samples, the temperature coefficients of 1-3L BN were −0.0187±0.0019, −0.0196±0.0013 and −0.0199±0.0006 cm^{-1}/K, respectively, very close to those of 1-3L BN suspended over SiO_2/Si.

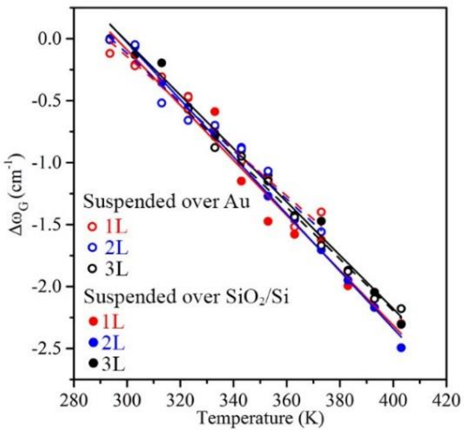

Fig. S3. Raman *G* band shifts of 1-3L BN suspended over Au/Si and SiO_2/Si as a function of temperature and the corresponding linear fittings.

Section S4. Absorbance of 1-3L BN measured on quartz

To estimate the 514.5 nm light absorbance of 1-3L BN, we also transferred BN nanosheets mechanically exfoliated from hBN single crystals on SiO_2/Si onto a transparent quartz substrate using the poly(methyl methacrylate) (PMMA) technique. To remove the PMMA, the BN nanosheets on quartz were heated at 550 °C in air for 3 h. fig. S4a shows the reflected optical microscopy image of a 1L BN on SiO_2/Si. fig. S4b and c show the digital photo of the quartz substrate and the reflected optical microscopy image of the 1L BN after transferred onto quartz and heated in air. Because of the small thickness of the 1L BN, AFM phase image was more effective than height image to visualize the transferred 1L BN on quartz (fig. S4d and e). An optical power meter (1916-C, Newport) was then used to measure the absorbance of the BN nanosheets under 514.5 nm laser. We used UV-Vis reflectance spectrum to estimate the reflectance of the bare quartz and quartz covered by few-layer BN produced by CVD, and quartz showed a nuance (i.e. slightly higher) in reflectance with or without the coverage by few-layer BN at 514.5 nm.

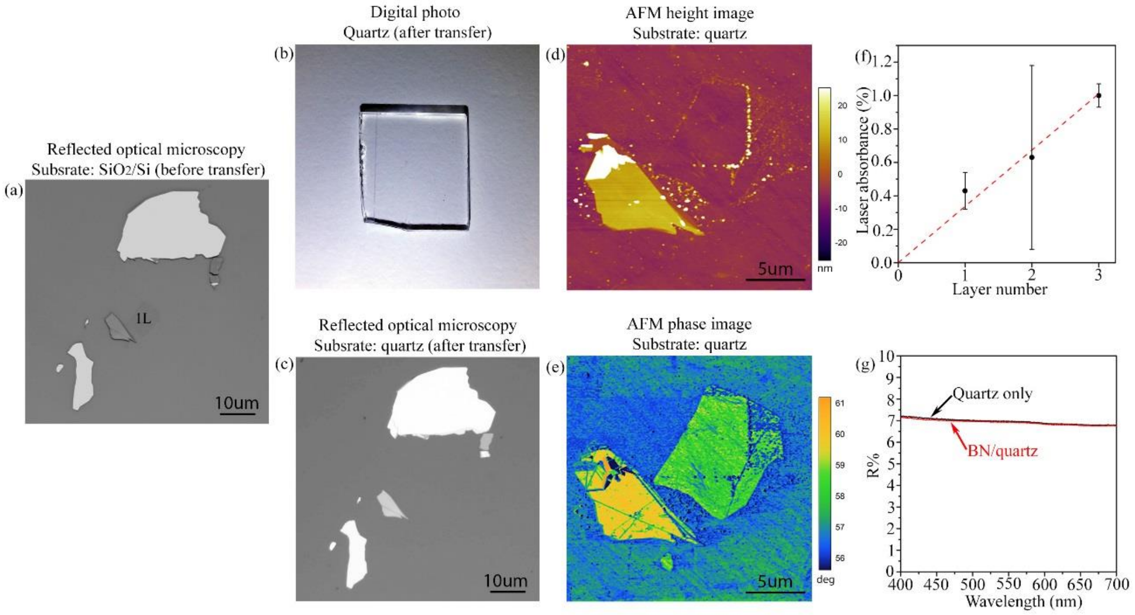

Fig. S4. Laser absorbance of atomically thin BN on quartz. (a) Reflected optical microscopy image of a 1L BN on SiO_2/Si substrate; (b) photo of the quartz substrate after the

transfer of BN and heat at 550 °C; (c) reflected optical microscopy image of the BN nanosheets on quartz; (d, e) AFM height and phase images of the BN on quartz; (f) light absorbance of 1-3L BN and the linear fitting; (g) UV-Vis reflectance spectrum of the bare quartz and quartz covered by CVD-grown few-layer BN (BN/quartz).

We also tried to use transmitted optical microscopy to estimate the absorbance of the 1L BN under visible light. fig. S5a shows that the 1L BN on quartz was not visible under the transmitted optical microscope, but the light intensity profiles of the dashed lines 1 and 2 did show small differences, as shown in fig. S5a and b. The absorbance of the 1L BN estimated from the profiles was ~0.4-0.5% in the visible range, in good agreement with the value measured by the optical power meter.

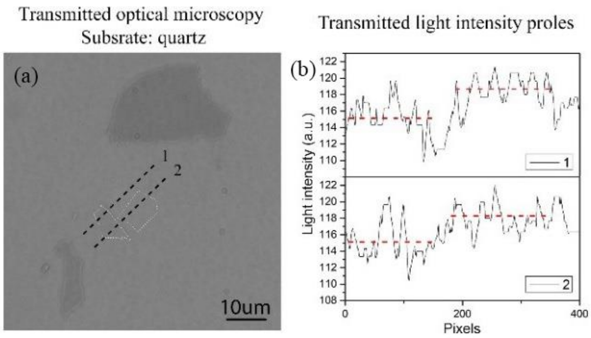

Fig. S5. Transmitted optical intensity of 1L BN. (a) Transmitted optical microscopy image of the same BN on quartz; (b) light intensity profiles of the dashed lines 1 and 2 in (a).

Section S5. Laser beam radius

The laser beam radius r_0 (objective lens 100×) was measured by performing a micro-Raman scan across the boundary of a partially Au-coated Si wafer. fig. S6a and b display the optical image of the boundary with and without Au coating and the corresponding Raman mapping

of the Si frequency at 520.5 cm^{-1} across the boundary. The measured intensity (I) was proportional to the total laser power incident on the sample. The step size used in the mapping was 0.1 μm. fig. S6c shows the distribution of I as a function of the distance (x) to the boundary. A Gaussian function $exp(-x^2/r_0^2)$ was used to fit the slope dI/dx to calculate r_0, which was found to be 0.31±0.01 μm (fig. S6d).

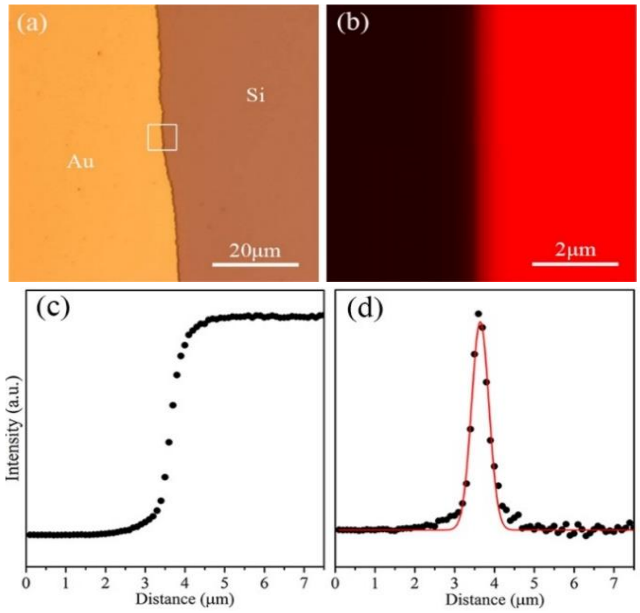

Fig. S6. Raman mapping of Si and corresponding fitting. (a) Optical microscopy image of the boundary with and without Au coating on a Si wafer used for the estimation of the laser beam radius; (b) Raman mapping of the Si band at 520.5 cm^{-1} in the squared area in (a); (c) distribution of the intensity of the Si Raman band across the boundary; (d) the corresponding Gaussian fitting.

Section S6. Error calculation

The thermal conductivity errors were calculated through the root sum square error propagation approach, where the temperature calibration by Raman peak position, the temperature resolution of the Raman measurement, and the uncertainty of the measured laser

absorbance were considered. For example, R=1.9 μm, a=(0.35±0.14)%, r_0=(0.31±0.01) μm, T_m=(302±3) K, P=1.1 mW, therefore $\kappa = \frac{\ln(\frac{R}{r_0})}{2\pi d \frac{Tm-Ta}{Q}} \alpha$ = ln(1.9/0.31)*0.35%*1.1*0.97*1000000/(2*3.14*

0.334*5) = 807 W/mK, and Error = $807 \times \sqrt{\left(\frac{0.01}{0.31}\right)^2 + \left(\frac{0.14}{0.35}\right)^2 + \left(\frac{3}{303}\right)^2}$ = 323, and then all the data in the similar temperature range, for example, 807±323 W/mK at 303K, 850±300W/mK at 300K, and 650±280 W/mK at 302 K, were averaged, and the error will be calculated by the root sum square error propagation approach: κ = (807+850+650)/3±$\sqrt{(323)^2 + (300)^2 + (280)^2}/3$ = (769±175)W/mK.

Section S7. Thermal conductivity of graphene as a control

For validation purpose, we used the same optothermal procedure to measure the thermal conductivity of monolayer graphene suspended over the same substrate with 3.8 μm holes connected by 0.2 μm wide trenches. The graphene sheets were mechanically exfoliated from highly oriented pyrolytic graphite (HOPG) using Scotch tape. The Raman results are shown in fig. S7. The calculated thermal conductivity of 1L graphene was 2102±221 W/mK.

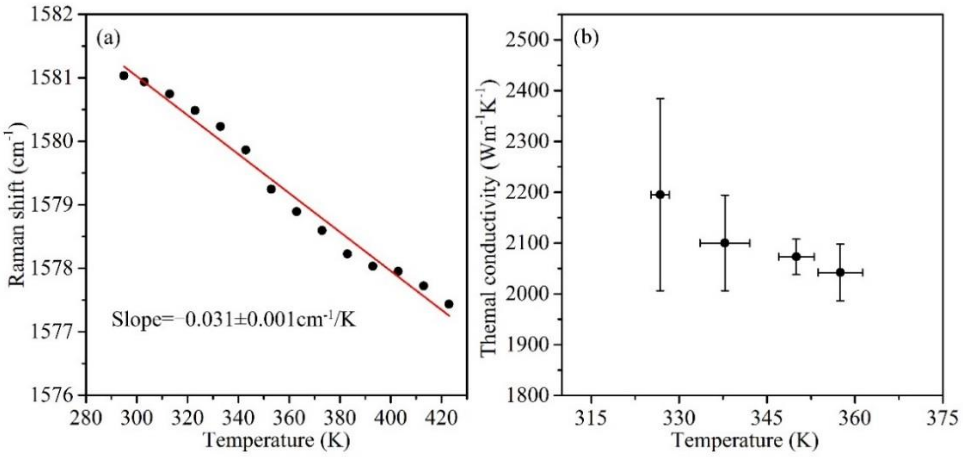

Fig. S7. The first-order temperature coefficient and thermal conductivity of graphene. (a) G band frequency of a monolayer graphene as a function of temperature, along with the corresponding linear fitting; (b) thermal conductivity of the graphene as a function of the Raman measured temperature.

Section S8. Thermal equilibration on MD simulations using LAMMPS

An initial equilibration using NVT ensemble on the systems was followed by the calculation of the thermal conductivity at the NVE ensemble as discussed in the main text.

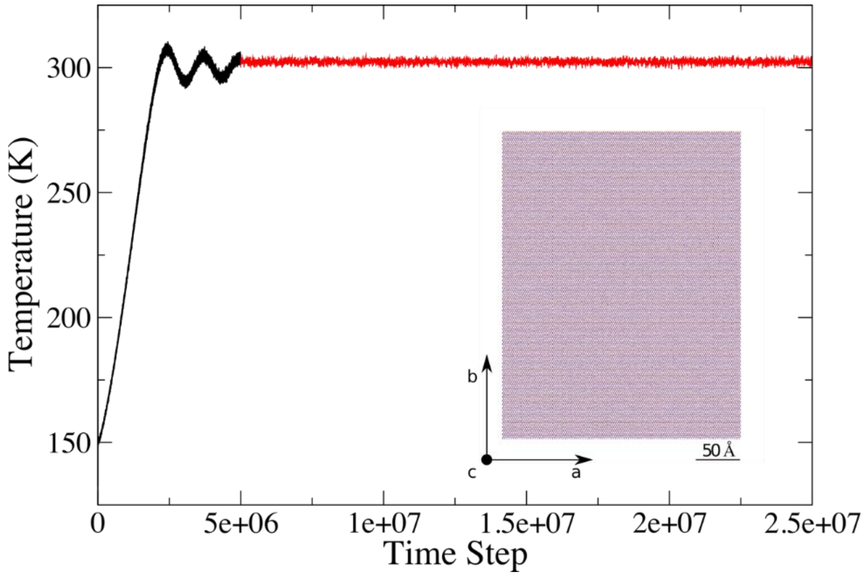

Fig. S8. Temperature versus time step for 1L BN. The black curve shows the initial equilibration using NVT ensemble, and the red curve is the subsequent evolution in NVE. A time step of 0.5 fs was used. See Methods for details.

Section S9. TEC of SiO_2/Si substrate simulated by FEM

Figure S9a shows the geometry model in Abaqus. The model was symmetric in both x-axis and y-axis. Only ¼ of the model was calculated for computational time efficiency. fig. S9b shows the dimension of the model projected on the x-y plane. The 90 nm top sheet was SiO_2 while the 810 nm bottom represented Si. Different thickness ratios between SiO_2 and Si were tested and numerically proved that the ratio of 9 was both computational accuracy and efficiency. Additionally, 8 nodes linear brick was used in this study. Element size was set to 30 nm. Different element sizes were also tested to prove accuracy. The simulation was conducted with Abaqus Standard implicit solver. The strain distribution of SiO_2/Si (fig. S9c) indicated the TEC of the top layer of 90 nm SiO_2/Si was ~60% of the TEC of Si, neither equal to the TEC of Si nor to that of SiO_2.

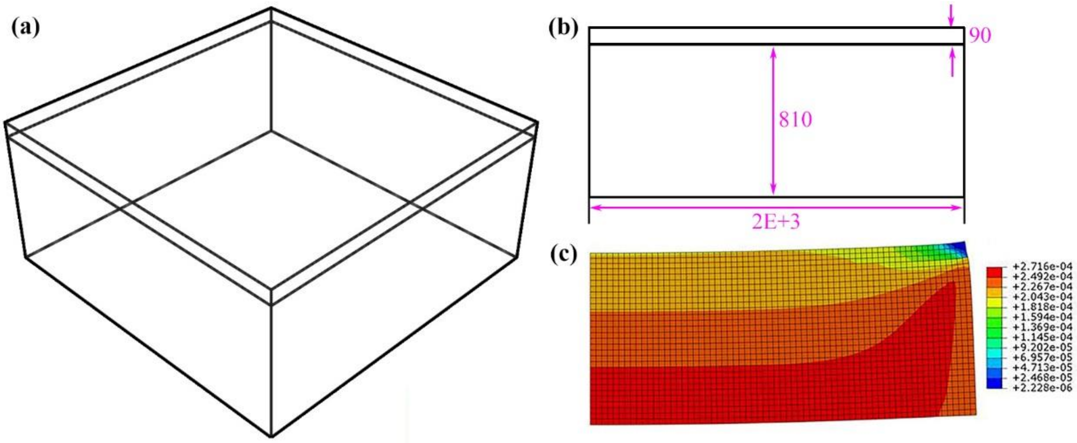

Fig. S9. Strain distribution of SiO_2/Si substrate. (a) Geometry model in finite element software Abaqus; (b) Dimension of modeled sheets (unit: nm); (c) strain distribution of 90 nm SiO_2/Si.

Section S10. Comparison of the TEC of common 2D materials

The thermal expansion coefficients of some common 2D materials at 300 K are compared in Table S1, with the values of BN from this study.

Table S1. TEC of 2D materials (10^{-6} K^{-1}).

2D materials	Monolayer	Bilayer	Trilayer
BN	-3.58±0.18	-2.55±0.28	-1.67±0.20
Graphene	-21.4±3.7	-10.9±2.5	-8.7±1.7
MoS_2	64.9±7.5	36.0±4.7	18.2±2.5
$MoSe_2$	106.2±6.4	54.4±3.5	34.6±2.8
WS_2	152.1±13.8	22.6±2.0	13.1±1.0
WSe_2	154.2±6.9	41.8±2.5	27.4±2.9
